# Angiotensin converting enzyme gene polymorphism in type II diabetics with nephropathy

**DOI:** 10.4103/0971-4065.59335

**Published:** 2009-10

**Authors:** V. V. S. Naresh, A. L. K. Reddy, G. Sivaramakrishna, P. V. G. K. Sharma, R. V. Vardhan, V. Siva Kumar

**Affiliations:** Department of Nephrology, Sri Venkateswara Institute of Medical Sciences, Sri Venkateswara University, Tirupati - 517 507, India; 1 Department of Biotechnology, Sri Venkateswara Institute of Medical Sciences, Sri Venkateswara University, Tirupati - 517 507, India; 2 Department of Statistics, Sri Venkateswara Institute of Medical Sciences, Sri Venkateswara University, Tirupati - 517 507, India

**Keywords:** Angiotensin converting enzyme gene polymorphism, nephropathy, type II diabetes mellitus

## Abstract

Nephropathy is an important and a frequent complication of long-term type II diabetic nephropathy. Strong evidence exists that genetic predisposition plays a major role in the development of diabetic nephropathy. Recent studies have implicated association between angiotensin converting enzyme (ACE) insertion/deletion (I/D) gene polymorphism and nephropathy. The deletion gene polymorphism of ACE gene has been shown to be associated with increased activity of this enzyme. This study examines the association of ACE I/D polymorphism with type II diabetes without nephropathy in 30 patients and type II diabetes with nephropathy in 30 patients. The results of the study suggest the association between the DD polymorphism and type II diabetes with nephropathy.

## Introduction

Chronic kidney disease (CKD) is a global threat to the health in general and for developing countries, in particular, because therapy is expensive and life-long. The estimated prevalence rate in CKD was 0.78% in India. If this data is applied to one billion population of India, there are approximately 7.85 million CRF patients in our country. Diabetes constitutes about 41% of the spectrum of the CKD.[1]

It is predicted that worldwide the prevalence of diabetes in adults would increase to 5.4% by the year 2024, from the prevalence rate of 4% in 1995. Consequently, the number of adults with diabetes in the world would rise from 135 million in 1995 to 300 million in the year 2025. While a 42% increase is expected in developed countries, a 170% increase is expected in the developing countries. Therefore, diabetic patients in the developing countries are even more vulnerable to develop the microvascular complications of diabetes including diabetic nephropathy.[2]

Strong evidence exists that genetic predisposition plays a significant role in the development of diabetic nephropathy in both type I and type II diabetes mellitus (DM).

Studies have shown that renin-angiotensin system may play an important role in the development of nephropathy in type II DM, and thus, the angiotensin converting enzyme (ACE) polymorphism may be a potential predictor for development of nephropathy in type II DM.[3,4]

ACE catalyses production of the vaso active peptide Angiotensin II from its precursor Angiotensin I. Within the diabetic kidney the effects of Angiotensin II include an increase of intraglomerular pressure and glomerular filtration rate. In addition to its hemodynamic effects Angiotensin II stimulates the production or release of several cytokine mediators of glomerulosclerosis such as Osteopontin, Platelet derived growth factor, Fibronectin and Transforming growth factor β.[3]

Genetic studies have revealed that the genes of renin angiotensin system (RAS) are highly polymorphic, raising the possibility that in addition to environmental factors, the genetic make up of RAS affects the status of RAS in individuals. One of such is the insertion/deletion (I/D) polymorphism of ACE gene. The ACE gene consists of 26 exons and spans 21 kb, on chromosome 17. Within intron 16, the polymorphism exists, consisting of the presence or absence of a 287 base-pair fragment.[5]

The deletion polymorphism is associated with elevated serum and cellular ACE levels. Earlier studies measuring plasma and tissue ACE levels demonstrated a significantly positive correlation between the D-allele and the RAS in normal subjects. It is observed that the II genotype has lowest ACE levels, the DD type has the highest and ID has the intermediate levels.[4–7]

The primary objective of the study was to find out pattern of distribution of ACE gene polymorphism in healthy controls, in patients of type II DM without nephropathy, in patients of type II DM with nephropathy and to study the relation between DD polymorphism and diabetes with nephropathy.

## Materials and Methods

This was a prospective study of two years duration (2006-2008) undertaken in our institute, after obtaining ethical clearance and patients consent to study the ACE gene polymorphism in type II DM patients with and without nephropathy. This study was carried out in three groups: normal controls (n = 30), type II diabetcis without nephropathy (n = 30), type II diabetics with nephropathy (n = 30) in our hospital during 2006-2008. This study was conducted after obtaining ethical clearance and patient consent. Adult patients with overnight fasting plasma glucose of more than 126 mg/dl on two consecutive days were included in type II diabetes mellitus (DM) category. Type II DM patients with 24 hours urinary protein of more than 500 mg with evidence of diabetic retinopathy were included in diabetic nephropathy group.

Determination of ACE genotype: DNA was isolated from 2 ml of whole-blood sample.[8] PCR amplification to detect ACE I/D polymorphism was carried out using the following primers: 5'-CTGGAGACCACTCCCATCCTTTCT-3' and 5'-GATGTGGCCATCACATTCGTCAGAT-3'. Amplification with this primer pair produces products of ∼490 and 190 bp corresponding to I and D alleles, respectively. Thermo cycling consisted of denaturation at 94°C for 60 S, annealing at 65°C for 60 S, and extension at 72°C for 90 S for 40 cycles followed by final extension at 72°C for seven minutes. PCR products (20 μl) were mixed with 4 μl 6X glycerol based gel loading buffer, size fractionated by electrophoresis 1% agarose gel that contained 0.5 mg/ml of ethidium bromide, and visualized by UV Tran illuminator. All D/D samples were amplified with an insertion-specific primer pair, which recognizes the inserted sequences, 5'-TGGGACCACAGCGCCCGCCACTAC-3' and 5'-TCGCCAGCCCTCCCATGCC CATAA-3'.[9,10] Cycling consisted of 40 cycles of denaturation at 94°C for 1 minute, and annealing/extension at 78°C for 1 minute, followed by a final extension at 72°C for 10 minutes.

Statistical analysis was done using SPSS software version 11.0. Genotype frequencies of ACE gene polymorphism were compared between type II diabetic patients with or without nephropathy using Chi square test. Genotypic and Allelic associations found significant by Chi square test were evaluated by computing odds ratio (OR) and 95% confidence intervals (CI). *P* values <0.05 were considered significant.

## Results

The demographic and other details of the subjects were summarized in Table 1. The ACE gene polymorphism analysis was summarized in Table 2. The D allele distribution was significantly increasesd in diabetes with nephropathy patients in comparison to controls. In the similar way DD genotype distribution was strongly increasesd in diabetes with nephropathy patients in comparison to controls. Odds ratio and 95% confidence intervals were shown amongst the groups in Table 3.

**Table 1:** Demographic and other clinical details

Variable	Normals (n = 30)	Dm without nephropathy (n = 30)	Dm with nephropathy (n = 30)
Age	33.57 ± 7.142	56.70 ± 6.314	54.47 ± 9.398
Sex (M/F)	15/15	16/14	16/14
Duration (year)	NA	12.83 ± 3.291	12.00 ± 2.274
BMI	25.657 ± 1.9648	27.240 ± 1.9042	28.503 ± 3.0792
Systolic BP	123.67 ± 7.489	141.73 ± 13.054	170.03 ± 18.446
Diastolic BP	80.07 ± 6.486	88.37 ± 8.344	99.53 ± 12.646

BMI: Body mass index

**Table 2:** Genotype and alleles distribution of angiotensin converting enzyme gene insertion/deletion polymorphism in Type 2 diabetes without nephropathy, Type 2 diabetes with nephropathy and control subjects

Geno type	Normal (%)	DM without nephropathy (%)	DM with nephropathy (%)	DM *vs*. DN	DM *vs*. Normals	DN *vs*. Normals
ID	17 (56.7)	11 (36.7)	11 (36.7)	*P* = 1.00	*P* = 0.121	*P* = 0.121
DD	1 (3.3)	7 (23.3)	15 (50.0)	*P* = 0.032	*P* = 0.023	*P* = 0.000
II	12 (40.0)	12 (40.0)	4 (13.3)	*P* = 0.020	*P* = 1.000	*P* = 0.020
Allele						
D	18 (29.0)	18 (29.0)	26 (41.9)	*P* = 0.020	*P* = 1.000	*P* = 0.000
I	23 (34.3)	23 (34.3)	15 (22.4)	*P* = 0.032	*P* = 0.023	*P* = 0.020

**Table 3:** Odds ratio and 95% confidence interval in the genotype and alleles distribution of angiotensin converting enzyme gene insertion/deletion polymorphism between the groups

Gene	Normals *vs*. DN	DM *vs*. DN	Normals *vs*. DM
			
	Odds ratio (95% CI)	Odds ratio (95% CI)	Odds ratio (95% CI)
II	0.231 (0.064, 0.831)	0.231 (0.064, 0.831)	1 (0.356, 2.809)
ID	0.443 (0.157, 1.247)	1 (0.350, 2.858)	0.443 (0.157, 1.247)
DD	29.00 (3.488, 241.13)	3.286 (1.085, 9.952)	8.826 (1.012, 76.960)
Allele			
I	0.034 (0.004, 0.287)	0.304 (0.1, 0.922)	0.113 (0.013, 0.988)
D	4.333 (1.203, 15.605)	4.333 (1.203, 15.605)	1 (0.356, 2.809)

## Discussion

The primary objective of the study was to find the pattern of distribution of ACE gene polymorphism in healthy controls, in type II DM without nephropathy, type II DM with nephropathy and to study the relation between DD gene polymorphism and diabetic nephropathy.

Although the data from Caucasian studies failed to confirm an increased risk for the development of diabetic nephropathy in IDDM and NIDDM being associated with D-allele, a role for this genetic marker in Asian patients with NIDDM cannot be ruled out.[11]

Grzeszczak *et al*. from Poland and Schmidt *et al*. from Germany did not find any association between the ACE gene polymorphism and nephropathy in NIDDM.[3,6] Yoshida *et al*. from Japan, Jeffers *et al*. from USA and Nikzamir *et al*. from Iran found a strong association betweezn ACE-DD genotype and/or D-allele and the risk for nephropathy in type II DM.[4,5,11,12]

In Indian studies, Viswanathan *et al*.[13] and Bhavani *et al*.[14] found a positive association between the D allele (ID and DD genotype) of the ACE polymorphism and diabetic nephropathy in south Indian type II diabetic patients.[13,14] Where as Ajay Kumar *et al*.[15] and Prasad P. *et al*.[16] found no relation between ACE gene polymorphism and development of diabetic nephropathy in type II diabetics in north Indian population.

Parving *et al*., reduction of end points in NIDDM with the Angiotensin II Antagonist Losartan (RENNAL) study found improvement in renal prognosis with Losartan in the D allele patients of NIDDM.[17]

In our study, a statistically significant relationship was observed between the D allele, DD genotype of the ACE polymorphism and diabetic nephropathy in South Indian type II diabetic nephropathy patients.
